# Pathohistological features of the aging human lacrimal gland

**DOI:** 10.3325/cmj.2023.64.307

**Published:** 2023-10

**Authors:** Koraljka Hat, Snježana Kaštelan, Ana Planinić, Danko Muller, Davor Ježek

**Affiliations:** 1 Department of Maxillofacial and Oral Surgery, University Hospital Dubrava, Zagreb, Croatia; 2 Department of Ophthalmology, University Hospital Dubrava, Zagreb, Croatia; 3 Department of Histology and Embryology, University of Zagreb School of Medicine, Zagreb, Croatia; 4 Clinical Department of Pathology and Cytology, University Hospital Dubrava, Zagreb, Croatia; 5 Department of Histology and Embryology, University of Zagreb School of Medicine, Zagreb, Croatia

## Abstract

**Aim:**

To assess sex-related differences in the pathohistological features of the human lacrimal gland and to investigate age- and sex-related differences in stereologically measured volume density of the secretory tissue, connective tissue, and fat.

**Methods:**

We performed an observational analysis of acinar atrophy, periacinar fibrosis, periductal fibrosis, ductal dilation, ductal proliferation, fatty infiltration, and lymphocyte infiltration of hematoxylin and eosin-stained lacrimal gland samples from 81 cornea donors. Stereological analysis of the volume density of the secretory tissue, connective tissue, and fat was performed on samples from 66 donors.

**Results:**

Up to 69% of all samples showed degenerative changes. Female samples had a higher frequency of all observed degenerative changes, except ductal dilation. While acinar atrophy was significantly more prevalent in women, ductal dilation was significantly more prevalent in men. Stereological analysis indicated lower portions of acini and higher portions of connective tissue and fat, as well as a more pronounced age-related progression of degenerative changes in female samples.

**Conclusion:**

Female lacrimal glands are more susceptible to degeneration, and this susceptibility could play an important role in the higher incidence of dry eye disease in older women. A further stereological analysis using more samples from younger age groups is needed to elucidate age-related and sex-related differences in the structure of the human lacrimal gland and their impact on dry eye disease.

A growing interest in the histological features of the lacrimal gland appeared in the middle of the 20th century, primarily because of its importance in maintaining the homeostasis of the tear film (TF). In recent decades, numerous animal models have been developed to clarify the physiology and pathophysiology of the lacrimal gland and its role in the development of dry eye disease (DED) (1-5). So far, few studies have been conducted using human lacrimal gland tissue (6-17).

Lacrimal glands are paired exocrine glands with a major role in the production of the middle aqueous layer of the TF (18-20). The majority of the aqueous layer is secreted from the lacrimal gland, while the lipid layer is produced by Meibom's glands (21). In humans, the lacrimal gland is situated in the superolateral part of the orbit and is incompletely divided into the orbital and palpebral lobe by a lateral part of the aponeurosis of the levator palpebrae superioris (LPS) muscle (22-24). Histologically, the lacrimal gland is a classic tubuloalveolar gland with a structure similar to salivary and mammary glands and the pancreas (25). It is also classified as a merocrine seromucinous exocrine gland (26). The glandular parenchyma consists of three main cell types: acinus cells, ductal epithelial cells, and myoepithelial cells (25). Traditionally, the secretory part of the lacrimal gland is considered to be the acinus, which consists of pyramid-shaped acinar cells, whose tips surround the central lumen. The islands of acini and smaller ducts form the lobules are surrounded by a thin layer of loose connective tissue. The canal system of the human lacrimal gland consists of small transitional (intercalated) ducts that merge into the interlobular ducts, which finally end in wide excretory ducts that open onto the surface of the eye (22,25,27). According to current findings in animal models, the canal system also has a significant secretory role (28).

Lacrimal glands, together with Meibomian glands, accessory lacrimal glands, cornea, conjunctiva, tear film, and interconnecting innervation constitute the lacrimal functional unit (LFU) (29,30). The LFU maintains a stable production, distribution, and clearance of the TF (31-33). DED can be caused by numerous intrinsic and extrinsic factors affecting any component of the LFU (34) mainly provoking systemic or local inflammation followed by discomfort or visual impairment (1,3,35-38). On the basis of the etiopathogenetic mechanisms, two entities can be distinguished: aqueous deficient dry eye, characterized by reduced tear production in the lacrimal glands, and evaporative dry eye, the most common type of DED, characterized by tear film instability as a result of Meiboimian gland dysfunction (39,40).

The prevalence of DED is increasing in both men and women with every decade of life over the age of 40 (41-44), with a greater prevalence in women than men at every age (41,42). Women have a 50%-70% higher risk of developing DED, with the difference being even more pronounced after menopause. This finding implicates that female sex and advanced age are the most important risk factors for developing DED (11,43). Sex-related differences in the prevalence of DED are largely attributed to the effects of sex hormones (androgens and estrogens), their receptors, the hypothalamus-pituitary-axis, corticosteroids, insulin, IGF-1 and pineal hormones, but also to sex chromosomes, sex-specific autosomal factors, and epigenetic factors (41,44).

Only a few pathohistological studies of the human lacrimal gland have been published so far. These studies observed age-related pathohistological changes, including a decrease in the weight of the gland, fibrosis and acinar atrophy, periductal fibrosis and dilatation of the ducts, interlobular ductal proliferation, and fatty and lymphocyte infiltration (6-10). Rare observations of sex-related differences in pathohistological findings suggested a higher frequency of focal adenitis in women, a significantly higher frequency of diffuse fibrosis and diffuse acinar atrophy in older women, and a significantly smaller surface area of the acini in female samples (6,9,11). To date, a detailed stereological analysis of the human lacrimal gland has not been performed.

The aim of this study was to analyze pathohistological features of the human lacrimal gland in a systematic manner, following criteria defined by Obata (6), and to obtain an insight into sex-related and age-related differences in the stereological features of the human lacrimal gland.

## Materials and methods

### Sample collection

Lacrimal gland samples for pathohistological analysis were collected from 81 cornea donors (34 female and 47 male) at the Eye Bank of the Zagreb University Hospital and Sestre Milosrdnice University Hospital Center, both in Zagreb, Croatia. Some of the samples were used in our previous study (44). The mean donor age was 67.58 ± 11.94 (range 26-89) years: 68.65 ± 12.41 years for female (range 36-89) and 66.8 ± 11.67 years for male donors (range 26-86). Stereological analysis was performed on lacrimal gland samples from 66 cornea donors (31 female and 35 male). The mean donor age was 67.26 ± 12.67 years (range 26-89): 68.52 ± 12.62 years (range 36-89) years for female donors and 66.14 ± 12.79 (range 26-86) years for male donors.

The inclusion criteria were explantation within 24 h from the moment of death and age above 18 years. The exclusion criteria were acute infection in the orbital area, systemic infection (sepsis, HIV, cytomegalovirus, hepatitis C, COVID-19), Sjögren’s syndrome, IgG4-related disease, inflammatory orbital pseudotumor, chronic graft vs host disease, sarcoidosis, insulin-dependent diabetes, previous surgery in the orbital area, radiation of the head and neck area, hormone replacement treatment, treatment with systemic corticosteroids, and hormone therapy for oncological patients. Most donors had both lacrimal glands explanted. In some cases, for objective reasons (damage to the cornea, severe bleeding after corneal explantation), only one gland was explanted. The glands were explanted with the transconjunctival approach. The procedure was carried out in compliance with asepsis and antisepsis measures. It started with a lateral commissurotomy. The gland was accessed transconjunctivally, through an incision in the lateral part of the upper fornix in the projection of the superolateral edge of the orbit. Then, a periosteal incision was made along the inner edge of the zygomatic process of the frontal bone above the lacrimal fossa. Following subperiosteal dissection and releasing of the lateral canthal ligament, the lateral aspect of the orbital lobe was approached. The orbital and then, in continuity with it, the palpebral lobe of the lacrimal gland were explanted. A suture was placed on the lateral commissure.

This study complied with the Declaration of Helsinki and was approved by the Ethics Committee of the University of Zagreb, School of Medicine; Ethics Committee of the University Hospital Center Zagreb; and Ethics Committee of the Sestre Milosrdnice University Hospital Center, Zagreb. Written informed consent was obtained from family members of all subjects involved in the study.

### Methods

Immediately after explantation, all tissue samples were transferred to a 10% neutral buffered formalin solution. The tissue was dehydrated, cleared, and embedded in paraffin. Five micrometer-thick sections of the tissue underwent standard hematoxylin and eosin staining.

Pathohistological analysis was performed with a light microscope Nikon Y100 (Nikon Instech Co. Ltd, Tokyo, Japan) at the magnification of 100 × . For each donor, we analyzed the surface area of 5 × 5 mm from one longitudinal section of the gland. Seven variables (acinar atrophy, periacinar fibrosis, periductal fibrosis, interlobular ductal proliferation, ductal dilation, lymphocytic infiltration, and fatty infiltration) were analyzed using the criteria established by Obata et al (6). Acinar atrophy and periacinar fibrosis were classified into four categories: not present (NP), focal (changes in only one lobule), lobular (changes present in less than 50% of the lobules), and diffuse (changes present in more than 50% of the lobules). Periductal fibrosis was classified as grade 0 (G0) if it was not present, grade 1 (G1) if it was present in less than 50% of the ducts, and grade 2 (G2) if it was present in more than 50% of the ducts. Lymphocytic infiltration was classified as grade 0 (G0) if it was not present, grade 1 (G1) if there was one focus of at least 50 lymphocytes, and grade 2 (G2) if two or more foci were present. Ductal proliferation, ductal dilation, and fatty infiltration were classified into two categories – present (P) or not present (NP). Fatty infiltration was defined as replacement of at least 30% of glandular parenchyma by adipose tissue, and was also classified as present (P) or not present (NP).

A quantitative histological analysis was performed on specimens from 35 male and 31 female lacrimal gland donors following the principles of classical stereology. We used a 42-point multipurpose test system according to Weibel built into the eyepiece of the Nikon Y100 light microscope (Nikon Instech Co. Ltd). Stereology allows a quantitative assessment of a three-dimensional structure of an object from its two-dimensional cross-section and can reliably detect differences only slightly more significant than the standard variations of 15%-20% (45,46). To calculate the required number of visual fields, an initial measurement was carried out on 5-10 test surfaces for each studied variable. From the obtained results, the arithmetic mean and standard deviation were calculated and included in the DeHoff equation: n = (20 × s/χ)^2^ (n = required number of visual fields, s = standard deviation, χ = mean).

The following stereological values were calculated:

1. volume density of the secretory tissue (Vvs) – the fraction of the secretory tissue in the volume unit (1 mm^3^) of the lacrimal gland at 40 × magnification;

2. volume density of the connective tissue (Vvc) – the fraction of the interlobular/interlobar connective tissue in the volume unit (1 mm^3^) of the lacrimal gland at 40 × magnification;

3. volume density of the fat tissue (Vvf) – the fraction of the fat tissue in the volume unit (1 mm^3^) of the lacrimal gland at 40 × magnification;

4. volume density of the intalobular acini (Vviac) – the fraction of the acini in the volume unit (1 mm^3^) of the glandular lobule at 100 × magnification;

5. volume density of the intralobular connective tissue (Vvic) – the fraction of the periacinar/periductal connective tissue in the volume unit (1 mm^3^) of the glandular lobule at 100 × magnification;

6. volume density of the intralobular fat tissue (Vvif) – the fraction of the fat tissue inside the volume unit (1 mm^3^) of the glandular lobule at 100 × magnification.

### Statistical analysis

The power of the test was calculated with a Fisher exact test. With the expected relative difference in the variables of at least 40%, the significance level α = 0.05, and the power of the test at 0.90, it was necessary to include at least 62 subjects, ie, 31 male and 31 female donors. The power analysis of the test was performed with the G*Power software, v. 3.1.3 (Heinrich Heine Universität, Düsseldorf, Germany).

Sex differences in the categorical variables were assessed with a χ^2^ test. The normality of the distribution of numerical data from stereological analysis was tested with a Kolmogorov-Smirnov test. To compare the sex differences in the measured variables, the Mann-Whitney U test was performed for the variables Vvs, Vvc, Vvic, and Vvif, and the *t* test was performed for independent samples Vvf and Vviac. Pearson’s parametric and Spearman's non-parametric correlation coefficients were calculated to assess the correlation of stereological parameters with age. *P* values lower than 0.05 were considered significant. Statistical analysis was conducted with GrahPad Prism 9.0 software (GraphPad Software Inc, San Diego, CA, USA).

## Results

### Frequency of pathohistological changes

**Figure 1:**
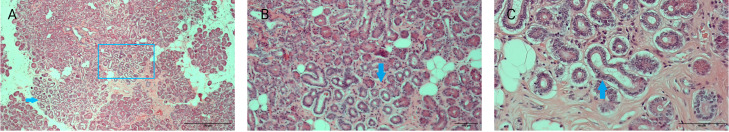
Acinar atrophy in a human lacrimal gland sample of a 59-year-old female donor. (**A**) A microphotograph of the glandular segment containing foci of acinar atrophy (blue arrow), which resemble excretory ducts. Framed in the blue window, the focus of acinar atrophy magnified in microphotograph B. Magnification 40 ×. (**B**) 100 × -magnified focus of acinar atrophy (blue arrow) highlighted in microphotograph A. (**C**) 200 × -magnified focus of acinar atrophy (blue arrow).

**Figure 2:**
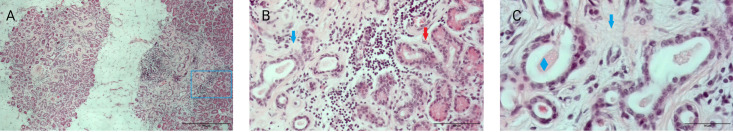
Advanced degenerative changes in a human lacrimal gland sample of a 73-year-old female donor. (**A**) Combination of advanced periacinar fibrosis and acinar atrophy accompanied by lymphocytic and fatty infiltration. Framed in the blue window, a segment of the gland containing a junction of one such area and relatively preserved acini within one lobule. Magnification 40 ×. (**B**) A magnified highlighted area from microphotograph A. On the left, severe periacinar fibrosis (blue arrow) surrounding atrophic acini, which resemble excretory ducts. Magnification 200 ×. On the right, relatively preserved acini (red arrow). Magnification 200 ×. (**C**) A detail of severe periacinar fibrosis (blue arrow) surrounding atrophic acini (blue rhombus). Magnification 400 ×.

**Figure 3:**
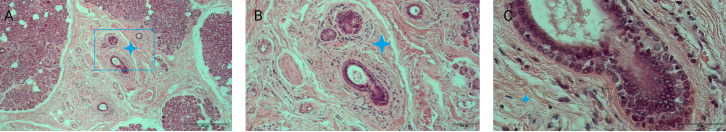
Periductal fibrosis in a human lacrimal gland of a 62-year-old male donor. (**A**) The interlobular area showing advanced fibrosis (blue cross) surrounding the excretory ducts and blood vessels. Framed in the blue window is a detail enlarged in images B and C. Magnification 40 ×. (**B**) A magnified highlighted area from microphotograph A showing abundant periductal connective tissue (blue cross). Magnification 100 ×. (**C**) A detail of the interlobular duct wall surrounded by thick connective tissue (blue cross). Magnification 400 ×.

**Figure 4:**
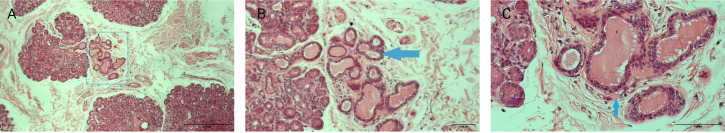
Ductal proliferation in a human lacrimal gland of an 83-year-old male donor. (**A**) Framed in the blue window, a segment of the gland containing an island of a multiplied interlobular duct. Magnification 40 ×. (**B**) A magnified highlighted area from microphotograph A showing proliferation of interlobular ducts (blue arrow). Magnification 100 ×. (**C**) A detail of multiplied interlobular ducts with amorphic content in the lumen. Magnification 200 ×.

**Figure 5:**
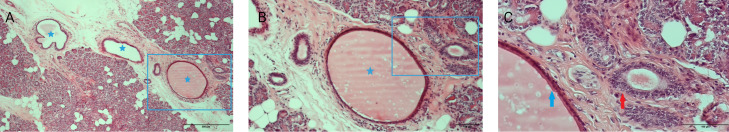
Ductal dilatation in a human lacrimal gland of a 65-year-old male donor. (**A**) Framed in the blue window is a segment of the gland showing dilated interlobular duct (blue star). Magnification 40 ×. (**B**) A magnified highlighted area from microphotograph A showing a dilated duct (blue star). Amorphic eosinophilic material in the duct presents stasis of tear fluid. Magnification 100 ×. (**C**) A detail of a typically thinned dilated duct wall (blue arrow) and a preserved interlobular duct (red arrow). Magnification 200 ×.

**Figure 6:**
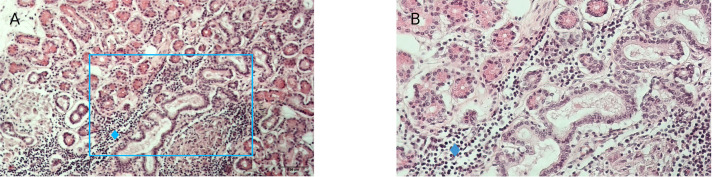
Lymphocytic infiltration in a human lacrimal gland of a 83-year-old female donor. (**A**) Framed in the blue window is a segment of the gland with dense periductal lymphocytic infiltration (blue rhombus). Magnification 100 ×. (**B**) A magnified highlighted area from microphotograph A showing lymphocytic infiltration (blue rhombus). Magnification 200 ×.

**Figure 7:**
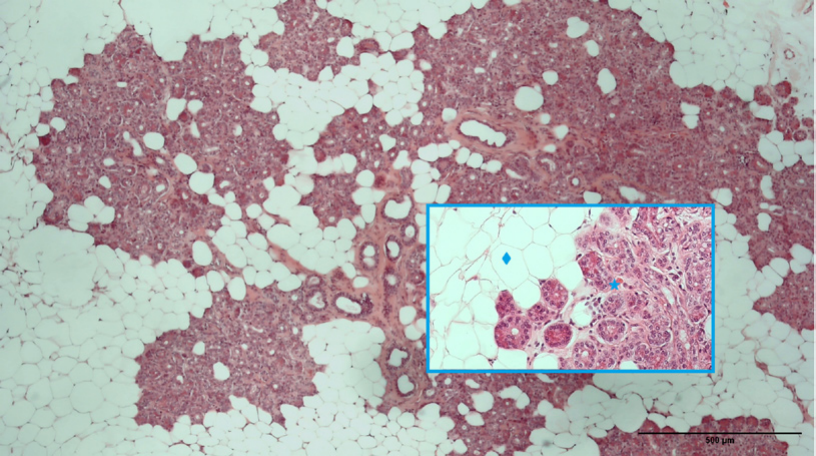
Fatty infiltration in a human lacrimal gland of a 64-year-old female donor. Magnification 40 ×. Framed in the blue window (200 × magnification) on the left side is the area of fatty infiltration (blue rhombus) and preserved acini on the right side (blue star).

**Figure 8:**
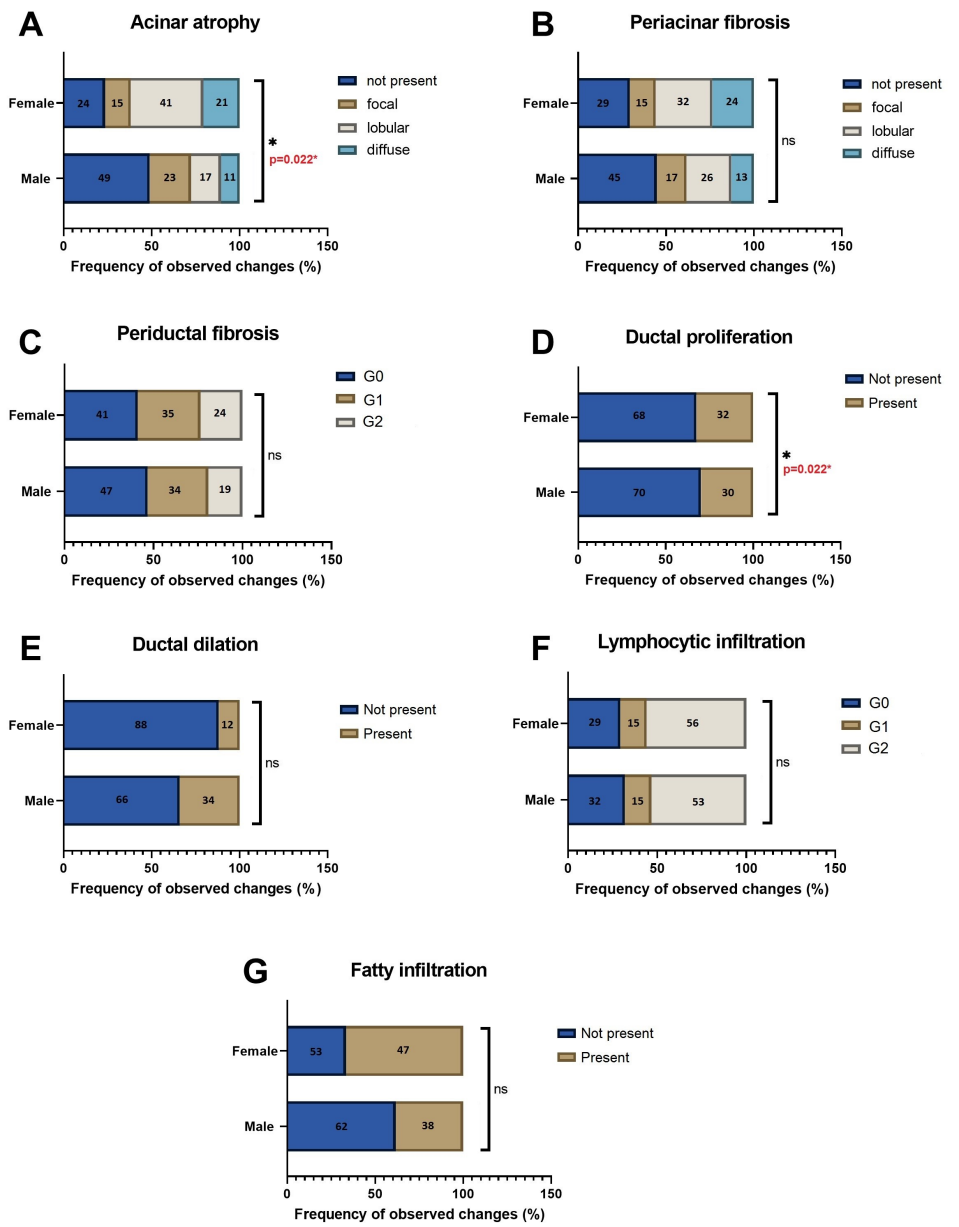
The frequency of pathohistological changes in the human lacrimal gland by sex (n = 81, f = 34, m = 47). (**A**) Acinar atrophy was significantly more frequent in female than male samples, Χ^2^ (3) = 9.64, P = 0.022. (**B**) There was no significant difference in the frequency of periacinar fibrosis between the sexes, Χ^2^ (3) = 2.91, P = 0.405. (**C**) There was no significant difference in the frequency of periductal fibrosis between the sexes, Χ^2^ (2) = 0.33, P = 0.805. (**D**) There was no significant difference in the frequency of ductal proliferation between the sexes, Χ^2^ (1) = 0.06, P = 0.805. (**E**) Ductal dilation was significantly more frequent in male than female samples, Χ^2^ (1) = 5.27, *P* = 0.022. (**F**). There was no significant difference in the frequency of lymphocytic infiltration between the sexes, Χ^2^ (2) = 0.07, P = 0.967. (**G**) There was no significant difference in the frequency of fatty infiltration between the sexes, Χ^2^ (2) = 0.62, P = 0.430.

A high percentage of the analyzed samples showed degenerative changes (Figure 1-7). Overall, the most frequently observed changes were lymphocytic infiltration (69%), acinar atrophy (62%), periacinar fibrosis (62%), and periductal fibrosis (56%). Female samples had a higher frequency of all observed degenerative changes except ductal dilation (Table 1, Figure 8).

**Table 1:** Results of pathohistological analysis for total sample, and male and female samples

		Total (n = 81)		Male (n = 47)		Female (n = 34)	
		N	%	N	%	N	%
**Acinar atrophy**	NP	31	38	23	49	8	24
	Focal	16	20	11	23	5	15
	Lobular	22	27	8	17	14	41
	Diffuse	12	15	5	11	7	21
**Periacinar fibrosis**	NP	31	38	21	45	10	29
	Focal	13	16	8	17	5	15
	Lobular	23	28	12	26	11	32
	Diffuse	14	17	6	13	8	24
**Periductal fibrosis**	G1	36	44	22	47	14	41
	G2	28	35	16	34	12	35
	G3	17	21	9	19	8	24
**Lymphocytic infiltration**	G0	25	31	15	32	10	29
	G1	12	15	7	15	5	15
	G2	44	54	25	53	19	56
**Ductal proliferation**	NP	56	69	33	70	23	68
	P	25	31	14	30	11	32
**Ductal dilation**	NP	61	75	31	66	30	88
	P	20	25	16	34	4	12
**Fatty infiltration**	NP	47	58	29	62	18	53
	P	34	42	18	38	16	47

*Abbreviations: P – present, NP – not present, G – grade.

### Sex-related differences in pathohistological features

While lobular and diffuse acinar atrophy were significantly more prevalent in women (χ^2^ (3) = 9.64, *P* = 0.022), ductal dilation was significantly more prevalent in men (χ^2^ (1) = 5.27, *P* = 0.022) (Figure 8).

No sex-related differences were observed for periacinar fibrosis (χ^2^ (3) = 2.91, *P* = 0.405), ductal proliferation (χ^2^ (1) = 0.06, *P* = 0.81), periductal fibrosis (χ^2^ (2) = 0.33, *P* = 0.805), fatty infiltration (χ^2^ (2) = 0.62, *P* = 0.430), and lymphocytic infiltration (χ^2^ (2) = 0.07, *P* = 0.967). The frequencies of each pathohistological feature according to sex are shown in Figure 8.

### Sex-related differences in the volume density of stereological parameters

**Figure 9:**
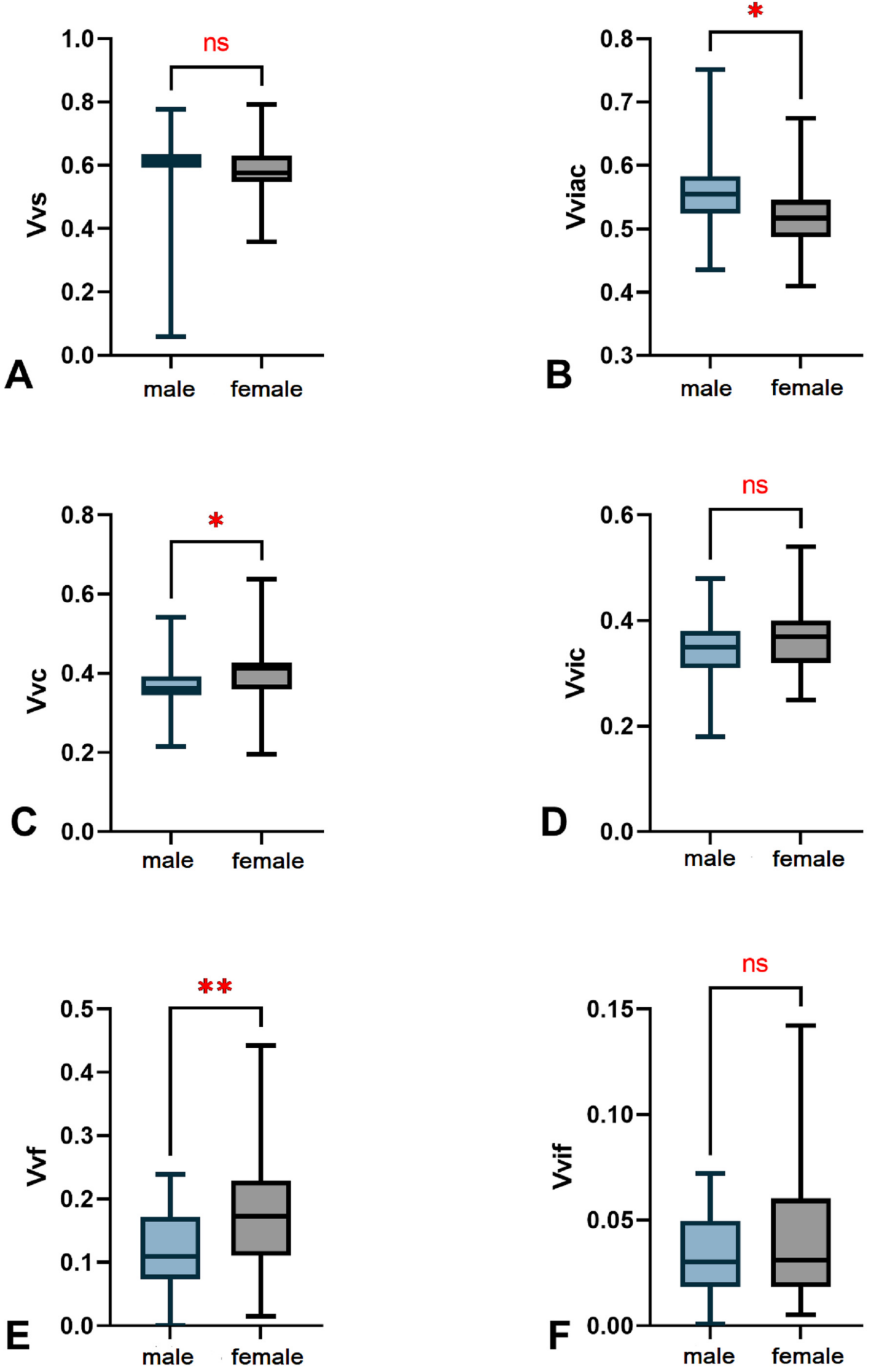
Sex-related differences in the stereological parameters. (**A**) No significant differences in the volume density of the secretory tissue (Vvs), *P* = 0.054. (**B**) Significant differences in the volume density of the intralobular acini (Vviac), *P* = 0.034. (**C**) Significant differences in the volume density of the connective tissue (Vvc), *P* = 0.012. (**D**) No significant difference in the volume density of the intralobular connective tissue (Vvic), *P* = 0.242. (**E**) Significant differences in the volume density of the fat (Vvf), *P* = 0.004. (**F**) No significant difference in the volume density of the intralobular fat (Vvif), *P < 0.05, **P < 0.01, ns-not significant.

In 66 samples of human lacrimal glands, male and female lacrimal glands significantly differed in Vvc (Z = -2.51; *P*=0.012), Vvf (*t* = 2.99; *P*=0.004), and Vviac (*t* = -2.16; *P* < 0.034), but not in Vvs (Z = -1.92; *P* = 0.053), Vvic (Z = -1.17, *P* = 0.242), and Vvif (Z = -0.78, *P* = 0.437) (Table 2). The volume density of acini per unit of lobule volume was significantly higher and volume density of connective and fatty tissue per unit volume of the lacrimal gland was significantly lower in male samples (Figure 9).

**Table 2:** Sex differences in the stereological parameters tested between male and female subjects

Volume density of	Male (n = 35)	Female (n = 31)	Test results	p
**Secretory tissue median** **(range)**	0.617 (0.718)	0.576 (0.434)	Z = -1.92	0.054*
**Connective tissue median** **(range)**	0.362 (0.327)	0.412 (0.442)	Z = -2.51	0.012*
**Fat tissue** **mean** **(standard deviation)**	0.123 (0.063)	0.175 (0.087)	*t* = 2.99	0.004†
**Intalobular acini mean** **(standard deviation)**	0.556 (0.058)	0.523 (0.064)	*t* = -2.16	0.034†
**Intralobular connective tissue median** **(range)**	0.355 (0.308)	0.373 (0.283)	Z = -1.17	0.242*
**Intralobular fat tissue** **median** **(range)**	0.030 (0.071)	0.031 (0.137)	Z = -0.78	0.437*

*Mann-Whitney U test.

‡t-test.

### Correlation of the volume density of stereological parameters with age

The correlation of individual stereological parameters with donor age was assessed for the total sample, and separately for male and female samples (Table 3).

**Table 3:** Spearman's and Pearson correlation coefficients for the volume density of stereological parameters and age

Volume density of	Total sample (n = 66)		Male (n = 35)		Female (n = 31)	
	r	p	r	p	r	p
**Secretory tissue**	r_s_ = -0.56	<0.0001	r_s_ = -0.36	0.04	r(31) = -0.66	<0.0001
**Connective tissue**	r_s_ = 0.53	<0.0001	r_s_ = 0.33	0.05	r(31) = 0.64	0.0001
**Fat tissue**	r_s_ = 0.31	0.01	r(35) = 0.14	0.41	r(31) = 0.39	0.03
**Intalobular acini**	r_s_ = -0.52	<0.0001	r(35) = -0.59	0.0002	r_s_ = -0.61	0.0003
**Intralobular connective tissue**	r_s_ = 0.36	0.003	r(35) = 0.54	0.0009	r_s_ = 0.34	0.06
**Intralobular fat tissue**	r_s_ = 0.11	0.39	r(35) = -0.03	0.87	r_s_ = 0.29	0.12

With increasing age of the donor, the volume density of secretory tissue per unit of gland volume and the volume density of acini per unit of lobule volume significantly decreased, ie, the volume density of connective tissue and fat per unit of gland volume and connective tissue per unit of lobule volume significantly increased.

When we looked at the male and female groups separately, the volume density of the secretory tissue per unit volume of the gland and the volume density of the acini per unit volume of the lobule significantly decreased with increasing age in both male and female subjects. The volume density of connective tissue and volume density of fat increased with age in female subjects, while no significant correlation was found in male subjects. The volume density of intralobular connective tissue significantly increased with age in male subjects. The volume density of intralobular adipose tissue did not significantly correlate with age in either group.

## Discussion

In this study, up to 76% of female and 68% of male glands showed some degree of degenerative changes. The most frequently observed changes were lymphocytic infiltration, acinar atrophy, periacinar fibrosis, and periductal fibrosis. Female samples had a higher frequency of all the observed degenerative changes except ductal dilation. A significant difference between sexes was observed only for acinar atrophy and ductal dilation. Stereological analysis did not show significant sex-related differences in the volume density of the total secretory tissue but indicated significantly higher volume density of the acini within the lobule of the male glands, as well as a significant negative correlation of the volume density of intralobular acini and secretory tissue with age in the total sample, and in male and female samples. The volume density of the connective tissue and the volume density of fat in the total volume of the gland were significantly higher in female glands and significantly positively correlated with age in female glands. The volume density of the intralobular connective tissue and fat did not show sex-related differences. The volume density of intralobular connective tissue positively correlated with age in male glands.

The results of our pathohistological analysis mostly agree with those of previous studies. The small number of previous studies conducted on human tissue mainly described age-related pathohistological changes, with only a few reporting sex differences. The most frequently observed pathohistological changes were decreased weight of the gland, fibrosis, acinar atrophy, ductal changes, and lymphocytic infiltration (6-8,10,12). The frequency of the observed pathohistological changes in our sample was similar to that in previous studies (10). Acinar atrophy is believed to start to occur even before middle age and progresses with age (8). This study showed that acinar atrophy was significantly more prevalent in women. This finding was also observed in one previous study (6), while others did not find significant sex-related differences in acinar atrophy (8). In our study, periacinar fibrosis was not sex dependent, which contradicts Obata's findings (6).

Some authors emphasized the role of ductal changes, speculating that repeated episodes of subclinical inflammation throughout life could obstruct the excretory ducts and lead to periductal fibrosis gradually extending proximally into the lacrimal lobules (8). The obstruction of ducts in the hilar region of the lobule, with subsequent dilation and tortuosity of ducts, sometimes as extreme as cystic formation, could cause secretion to stagnate and affect the secretory part of the gland in terms of acinus atrophy. Focal occurrence of observed changes could explain the variations in the degree of acinar atrophy between the lobules (8). The predominance of the interlobular ductal dilation in the palpebral lobes suggests obstruction of tear outflow in the conjunctival fornix (6). Contrary to previous studies, we found a significantly higher frequency of ductal dilation in male glands, perhaps due to an uneven fraction of palpebral and orbital lobes in the samples. Other categories of ductal changes failed to show sex-related differences in our study, probably because of the high median age of both donor groups. Lymphocytic infiltration was observed in 69% of all samples, without sex differences in its frequency, which is in accordance with previous findings (8,12). Fatty infiltration was observed in 42% of all samples. This percentage is slightly higher than in a previous study (6) and could be explained by a difference in the median age of patients. No sex-related differences in the frequency of fatty infiltration were found.

Stereology provides an objective insight into tissue morphology. We found a nonsignificant sex difference in the volume density of the secretory tissue, but significantly lower volume density of the acini in female samples. These findings suggest a more preserved secretory potential of the lacrimal gland in older men compared with older women. Cornell-Bell et al performed the only stereological study of the human lacrimal gland published so far. They measured the surface area of 50 acini in five human lacrimal glands and found that men had 21% larger acinar surface than women (11), a finding that agrees with our results. The main limitation of the study by Cornell-Bell et al is a small sample size with only five glands per sex. The same authors, using the same methodology, measured the diameter of the acinus for rats, mice, guinea pigs, and rabbits. The results indicated a significant difference in the surface of the acini between the studied species, suggesting a link between sexual dimorphism and androgen levels. Their results are not comparable with the results of our study since they performed the surface density analysis but did not measure the volume density. Hamid et al measured the mean diameter of the acinus in female glands of prereproductive, reproductive, and postreproductive age, finding that the number of acini was lowest in postreproductive age and the highest in reproductive age (47).

As expected, in our study, the volume densities of the secretory tissue and intralobular acini were negatively correlated with age in both sexes, a result indicating that the portion of the secretory tissue decreased with age in both men and women. These findings agree with previously described age-dependent glandular weight decrease, increased fibrosis, and acinar atrophy in human lacrimal glands (47).

In our study, the portions of connective tissue and fat in the total volume of the gland were significantly higher in female glands, but in both sexes they increased with age, so that the portions of the secretory and non-secretory tissue of the gland were inversely proportional to age. In male glands, the positive correlation of the portion of the connective tissue and fat in the total volume of the gland with age did not reach statistical significance, which suggests that these changes are less intensive in men. A previous study (8) has shown that acinus atrophy and fibrosis begin even before middle age, although they are much more pronounced in older age. This study however did not explore sex differences (8).

The volume densities of the intralobular connective tissue and fat were not significantly different between the sexes. Interestingly, the volume density of the intralobular connective tissue significantly positively correlated with age in male samples, which should be further investigated. Intralobular fat infiltration did not show any age-related or sex-related differences, as opposed to interlobular/interlobar fat infiltration, a finding suggesting its insignificance in the spectrum of common age-related degenerative changes of the human lacrimal gland.

The main limitation of this study was the high median donor age (67.58±11.94). Considering only four female and five male glands from donors under the age of 55 in the total sample of 66 glands, our study could not provide reliable stereological values for the donors of reproductive age. Future research should include more participants of reproductive age. Another limitation is the lack of ductal system analysis. The ductal system could not be analyzed with stereological analysis due to its high variability and extremely high number of visual fields required for analysis. An additional limitation is that we could not evaluate signs and symptoms of DED in participants, since the samples were collected *post-mortem*. The pathohistological analysis has its limitations, not only because it is an observational analysis, but also because of the heterogeneity of the samples in terms of expressed degenerative changes (6).

In conclusion, this study presents the first published confirmation of sexual dimorphism in the volume density of the acini, connective tissue, and adipose tissue in the human lacrimal gland. Lower volume density of the acini and higher volume density of fat and connective tissue, as well as more pronounced increase in degenerative changes in aging female glands, indicate a greater susceptibility of female lacrimal glands to degeneration. This susceptibility could be related to a higher incidence of DED in older women, which was already extensively described (41,42,48-51). A further stereological analysis of more samples from younger age groups is needed to elucidate age-related and sex-related differences in the structure of the human lacrimal gland and their impact on DED.
